# Inhibition of glycolysis disrupts cellular antioxidant defense and sensitizes HepG2 cells to doxorubicin treatment

**DOI:** 10.1002/2211-5463.12628

**Published:** 2019-04-11

**Authors:** Agnieszka Korga, Marta Ostrowska, Magdalena Iwan, Mariola Herbet, Jaroslaw Dudka

**Affiliations:** ^1^ Independent Medical Biology Unit Medical University of Lublin Poland; ^2^ Department of Toxicology Medical University of Lublin Poland

**Keywords:** 2‐deoxyglucose, 3‐promopyruvate, dichloroacetate, doxorubicin, glycolysis inhibitors, oxidative stress

## Abstract

Increased glucose consumption is a known hallmark of cancer cells. Increased glycolysis provides ATP, reducing agents and substrates for macromolecular synthesis in intensely dividing cells. Therefore, inhibition of glycolysis is one strategy in anticancer therapy as well as in improved efficacy of conventional anticancer chemotherapeutic agents. One such agent is doxorubicin (DOX), but the mechanism of sensitization of tumor cells to DOX by inhibition of glycolysis has not been fully elucidated. As oxidative stress is an important phenomenon accompanying DOX action and antioxidant defense is closely related to energy metabolism, the aim of the study was the evaluation of oxidative stress markers and antioxidant abilities of cancer cells treated with DOX while glycolysis is inhibited. HepG2 cells were treated with DOX and one of three glycolysis inhibitors: 2‐deoxyglucose, dichloroacetate or 3‐promopyruvate. To evaluate the possible interaction mechanisms, we assessed mRNA expression of selected genes related to energy metabolism and antioxidant defense; oxidative stress markers; and reduced glutathione (GSH) and NADPH levels. Additionally, glutamine consumption was measured. It was demonstrated that the chemotherapeutic agent and glycolysis inhibitors induced oxidative stress and associated damage in HepG2 cells. However, simultaneous treatment with both agents resulted in even greater lipid peroxidation and a significant reduction in GSH and NADPH levels. Moreover, in the presence of the drug and an inhibitor, HepG2 cells had a reduced ability to take up glutamine. These results indicated that cells treated with DOX while glycolysis was inhibited had significantly reduced ability to produce NADPH and antioxidant defenses.

**Abbreviations**
2‐DG: 2‐deoxyglucose
3‐BP: 3‐bromopyruvate
4‐HAE: 4‐hydroxyalkenals
AP: abasic
DCA: dichloroacetate
DOX: doxorubicin
GLUT1: glucose transporter 1
GPX: glutathione peroxidase
GSH: reduced glutathione
HK: hexokinase
LDH: lactate dehydrogenase
LPO: lipid peroxidation
MDA: malondialdehyde
MTT: 3‐(4,5‐dimethylthiazol‐2‐yl)‐2,5‐diphenyltetrazolium bromide
NNT: nicotinamide nucleotide transhydrogenase
OXPHOS: mitochondrial oxidative phosphorylation
PCK2: phosphoenolpyruvate carboxykinase 2
PDK: pyruvate dehydrogenase kinase
PPIA: peptidylprolyl isomerase A
qRT‐PCR: quantitative real‐time PCR
ROS: reactive oxygen species
SOD: superoxide dismutase

In the recent years, glycolysis inhibition has become one of the most important strategies in anticancer therapy. Even though the fact that cancer cells show increased glycolysis has been known since the 1920s, recent decades have brought an explanation of the biological significance of this phenomenon. It has become clear that intense glycolysis, even under oxygen availability (aerobic glycolysis; the Warburg effect), provides ATP, reducing agents (NADPH) and substrates for nucleic acid, lipid and amino acid synthesis in the intensely dividing cells 1, 2, 3. Upregulated glucose metabolism is applied in the sensitive detection of malignant lesions in the fluorodeoxyglucose positron emission tomography scan 4. Since mitochondrial oxidative phosphorylation (OXPHOS) is suppressed in cancer cells, glycolysis becomes their main source of ATP. For this reason, inhibition of glycolysis can selectively deprive cancer cells of ATP and biomass, leaving normal cells that produce ATP in the tricarboxylic acid cycle unharmed 5, 6, 7. The targeted enzymes include hexokinase (HK, EC:2.7.1.1), phosphofructokinase (EC:2.7.1.11), pyruvate kinase (EC:2.7.1.40), lactate dehydrogenase (LDH, EC:1.1.1.27) and pyruvate dehydrogenase kinase (PDK, EC:2.7.11.2). Various small molecule inhibitors of glycolytic enzymes exhibit significant anticancer activity in both *in vitro* and *in vivo* studies 8. Most of them are in the preclinical phase; however, some of them, e.g. 2‐deoxyglucose (2‐DG) and dichloroacetate (DCA), have already been tested in clinical trials. Apart from the fact that glycolysis inhibitors are toxic to the cancer cells, they have also been shown to enhance the effects of conventional chemotherapeutic agents, especially against the cancer cells with defective mitochondria or under hypoxic conditions 9. One hypothesis assumes that inhibition of glycolysis deprives cancer cells of cellular ATP and simultaneously deactivates the ATP‐binding cassette transporters responsible for multidrug resistance 10. In recent years, there have been several reports on sensitization of tumor cells to doxorubicin (DOX) by inhibiting glycolysis; however, the mechanism of this phenomenon has not been fully elucidated yet 9, 10, 11.

It is believed that DOX exerts its anticancer effect via two different pathways. The primary mechanism of action of DOX involves the drug's ability to intercalate within the DNA base pairs causing breakage of the DNA strands and inhibition of both DNA and RNA synthesis. DOX inhibits the enzyme DNA topoisomerase II (EC:5.99.1.3), causing DNA damage and induction of apoptosis 12. The other mechanism relies on generation of reactive oxygen species (ROS) by DOX, which causes cell death in both cancer and normal cells. Although oxidative stress is more often considered in the context of anthracycline cardiotoxicity 13, 14, it also plays a significant role in cancer cells, which typically present a higher level of ROS than normal cells 15. Increased ROS and oxidative damage are pivotal in increasing the mutation rate, activating oncogenes, enhancing metabolic reprogramming and tumor progression 16. Oxidative stress also changes the tumor's microenvironment, i.e. in order to obtain nutrients and promote growth, hydrogen peroxide produced by the tumor tissue can initiate destruction of the normal surrounding tissue 17. Because of this sharp reliance on ROS production, cancer cells are more vulnerable to further disturbance of their redox status than normal cells 18. Thus, due to the enhanced antioxidant capacity of tumors, a therapeutic strategy using this phenomenon should be based not only on ROS production but also on inhibition of the antioxidant defense system 19. Glycolysis inhibitors, especially HK inhibitors, may act in this way as they block the flow of the main substrate into the pentose phosphate pathway. By depriving the cell of NADPH, they limit its reducing power and antioxidant defense. For this reason, we hypothesized that oxidative stress may play a crucial role in the synergistic effect of glycolysis inhibition and DOX treatment. In this study, we evaluated the effect of DOX on HepG2 cells simultaneously treated with 3‐bromopyruvate (3‐BP; HK inhibitor), 2‐DG (HK inhibitor) or DCA (PDK inhibitor).

Glycolysis inhibitors form a heterogeneous group of compounds with a multidirectional activity. To the best of our knowledge, this study is the first to compare the synergistic interactions of DOX with various inhibitors in cancer cells; we also attempt to pinpoint the common, universal mechanism of this phenomenon in relation to redox imbalance and antioxidant defense. Exploring these mechanisms is essential in considering the use of a glycolysis inhibition strategy to sensitize tumor cells to DOX.

## Methods

### Cell culture and treatment

The culture of human hepatocellular carcinoma cells (HepG2, HB‐8065) (ATCC, Manassas, Virginia, USA) was performed in Eagle's minimum essential medium (ATCC) supplemented with 10% fetal bovine serum (Life Technologies, Carlsbad, California, USA). The cells were incubated at 37 °C with 5% CO_2_ in an air atmosphere. The tested cells were incubated for 48 h with 1 μm DOX (EBEWE Pharma, Unterach, Austria) and 2 mm of 2‐DG (Sigma‐Aldrich, USA), 10 μm of 3‐BP (Sigma‐Aldrich, Saint Louis, Missouri, USA) and 1 mm of DCA (Sigma‐Aldrich) or combined (DOX + single glycolysis inhibitor). On the basis of a preliminary study, the lowest concentration of each inhibitor that revealed the synergistic effect with DOX was chosen. The concentration of DOX was based on reported clinically achievable plasma concentrations 14 and cytotoxicity observed for HepG2 cells (IC_50_).

### Cytotoxicity analyses

Cytotoxicity was evaluated with the 3‐(4,5‐dimethylthiazol‐2‐yl)‐2,5‐diphenyltetrazolium bromide (MTT) assay using the MTT Assay Kit (Life Technologies). The test is based on living cells’ ability to reduce the orange tetrazolium salt to water‐insoluble purple formazan crystals. The cells were inoculated into a 96‐well plate at a concentration of 2.5 × 10^5^ cells·mL^−1^. The tested compounds were added when 70–80% confluence was achieved. The MTT solution (4.0 mg·mL^−1^) was added to the culture 48 h after the chemicals. Following a 4‐h incubation, the medium with MTT was removed and the formed crystals were dissolved in DMSO. The solution absorbance was measured at 540 nm with a PowerWave™ microplate spectrophotometer (Bio‐Tek Instruments, Winooski, Vermont, USA). Each experiment was conducted three times with measurement in triplicate.

### Apoptosis and necrosis detection

Quantification of apoptosis and necrosis was conducted using the Annexin V Apoptosis Assay (Chemometec., Lillerød, Denmark) and in compliance with the manufacturer's recommended protocol. The cells were inoculated into a six‐well plate at a concentration of 2.5 × 10^5^ cells·mL^−1^. The tested compounds were added when 70–80% confluence was achieved. After a 48‐h incubation, the cells were washed with PBS and detached using 0.25%/EDTA trypsin (Corning, Corning, New York, USA). Next, the cells were centrifuged at 400 ***g*** for 5 min and then resuspended in the annexin V binding buffer and stained with the annexin V and Hoechst 33342. After a 15‐min incubation at 37 °C, the stained cells were spun down and resuspended in 100 μL annexin V binding buffer supplemented with ethidium homodimer III and immediately analyzed. The analysis was performed by image cytometry with a Nucleo Counter NC3000 (Chemometec).

### Quantitative real‐time PCR analysis

The quantitative real‐time PCR (qRT‐PCR) method was used to evaluate the expression of selected genes in the HepG2 cell line. The cells were inoculated into a six‐well plate at a concentration of 2.5 × 10^5^ cells·mL^−1^. The tested compounds were added when 70–80% confluence was achieved. After 24‐ and 48‐h incubation, the cells were harvested with trypsin. RNA was isolated from the cells using the Syngen Blood/Cell RNA Mini Kit (Syngen Biotech, Wroclaw, Poland). All samples of good quality (*A*_260/280_ ratios approximately 2.0) were reversely transcribed with the use of random primers and NG dART RT‐PCR reagents (EURx, Gdansk, Poland) as recommended by the manufacturer. The relative mRNA expression level was determined by the qPCR and the ΔΔ*C*_t_ method using *18S ribosomal RNA* (*RNA18SN5*) and *peptidylprolyl isomerase A* (*PPIA*) as endogenous controls. The reference genes were selected on the basis of our preliminary studies, where *RNA18SN5* and *PPIA* were the most stable reference genes in the HepG2 cells and they remained unaffected by the experimental conditions. The reaction was carried out in quadruplicate using the SmartChip Real‐Time PCR System (Wafergen Biosystems, Fremont, California, USA) and SG qPCR Master Mix (2×) (EURx) according to the manufacturer's instructions. The primer sequences are listed in Table 1. Data quality screening based on amplification curves and *C*_t_ values was performed to remove any outlier data before ΔΔ*C*_t_ calculations and to determine the fold change in mRNA levels. Statistical analysis was performed on RQ values (RQ = $2^{-\Delta \Delta C_{t}}$).

**Table 1:** Primer sequences with gene symbols, protein names and GenBank reference sequence accession numbers

Gene symbol	Protein name	RefSeq ID	Forward sequence (5′ → 3′)	Reverse sequence (5′ → 3′)
*GLUT1*	Solute carrier family 2 member 1	NM_006516	TCACTGTGCTCCTGGTTCTG	CCTGTGCTCCTGAGAGATCC
*HK2*	Hexokinase 2	NM_000189	TAGGGCTTGAGAGCACCTGT	CCACACCCACTGTCACTTTG
*LDHA*	Lactate dehydrogenase A	NM_005566	ACTGCAAACTCCAAGCTGGT	CGCTTCCAATAACACGGTTT
*PCK2*	Phosphoenolpyruvate carboxykinase 2, mitochondrial	NM_001018073.2	GGGTGCTAGACTGGATCTGC	CTGGTTGACCTGCTCTGTCA
*PDK1*	Pyruvate dehydrogenase kinase 1	NM_001261816.1	CACGCTGGGTAATGAGGATT	ACTGCATCTGTCCCGTAACC
*NNT*	Nicotinamide nucleotide transhydrogenase	NM_012343.3	ATTGGTGGCGTCACCTTTAG	CACCCATGACAGCAGAGAGA
*SOD2*	Superoxide dismutase 2	NM_000636	CTTCAGGGTGGTATGGCTGT	TGGCCAGACCTTAATGTTCC
*GPX1*	Glutathione peroxidase 1	NM_000581	TTGACATCGAGCCTGACATC	ACTGGGATCAACAGGACCAG
*RNA18SN5*	18S ribosomal N5	NR_003286	GAAACTGCGAATGGCTCATTAAA	CACAGTTATCCAAGTGGGAGAGG
*PPIA*	Peptidylprolyl isomerase A	NM_021130	TTCATCTGCACTGCCAAGAC	TCGAGTTGTCCACAGTCAGC

### Oxidative stress detection

The CellROX^®^ Green Reagent (Invitrogen, Carlsbad, California, USA) was used for oxidative stress detection. CellROX is a fluorogenic probe which is weakly fluorescent while in a reduced state. It exhibits bright green photostable fluorescence upon oxidation by ROS and subsequent binding to DNA, with the absorption/emission maxima of 485/520 nm.

The cells were inoculated into a six‐well plate at a concentration of 2.5 × 10^5^ cells·mL^−1^. The tested compounds were added when 70–80% confluence was achieved. After a 48‐h incubation, the cells were stained with the CellROX Orange Reagent and Hoechst 33342 in complete medium for 30 min at 37 °C. Next, the cells were washed with PBS and the fluorescence signal was measured by means of the SpectraMax i3 Multi‐Mode Platform (Molecular Devices, San Jose, California, USA). Each experiment was conducted three times with measurement in triplicate.

### Determination of DNA oxidative damage

The cells were inoculated into a six‐well plate at a concentration of 2.5 × 10^5^ cells·mL^−1^. The tested compounds were added when 70–80% confluence was achieved. After a 48‐h incubation DNA was isolated with the Syngen DNA Mini Kit (Syngen) in compliance with the manufacturer's protocol. The concentration and purity of the genomic DNA were measured with the MaestroNano Micro‐Volume Spectrophotometer (Maestrogen Inc., Hsinchu City, Taiwan) and adjusted to 100 μg·mL^−1^ in Tris‐EDTA buffer. The oxidative DNA damage was evaluated by measuring the number of abasic sites (AP sites) with a DNA Damage Quantification Kit (Dojindo, Kumamoto, Japan) following the manufacturer`s instructions. ROS oxidative attacks on the deoxyribose moiety lead to the release of free bases from DNA, thereby generating strand breaks with various sugar modifications and simple AP sites. The aldehyde‐reactive probe *N*′‐aminooxymethylcarbonylhydrazin‐d‐biotin reacts specifically with the aldehyde group present on the open ring form of AP sites, enabling detection of the DNA modifications that result in the formation of an aldehyde group. A biotin–avidin‐specific connection and horseradish peroxidase were used for colorimetric detection at 650 nm performed with the use of the PowerWave™ microplate spectrophotometer (Bio‐Tek Instruments). Each experiment was conducted three times with measurement in triplicate.

### Lipid peroxidation

The lipid peroxidation (LPO) level evaluation was based on the measurement of malondialdehyde (MDA) and 4‐hydroxyalkenals. The assay was conducted in compliance with the manufacturer's instructions (OxisResearch, Burlingame, California, USA). The cells were inoculated into 75 cm^2^ culture flasks at a concentration of 2.5 × 10^5^ cells·mL^−1^. The tested compounds were added when 70–80% confluence was achieved. After a 48‐h incubation, the cells were washed and a reaction of a chromogenic reagent, *N*‐methyl‐2‐phenylindole (R1), with MDA was conducted at 45 °C in the obtained extracts. One molecule of MDA or 4‐hydroxyalkenals (4‐HAE) reacts with two molecules of the R1 reagent to yield a stable chromophore. The final readings were made using a Power Wave Microplate Spectrophotometer (Bio‐Tek) at 586 nm. Each experiment was conducted three times with measurement in triplicate.

### NADPH concentration

The NADPH concentration was determined by the spectrophotometric method described in the commercial kit NADP/NADPH Quantitation Colorimetric Kit (BioVision, Milpitas, California, USA). The cells were inoculated into 75 cm^2^ culture flasks at a concentration of 2.5 × 10^5^ cells·mL^−1^. The tested compounds were added when 70–80% confluence was achieved. After a 48‐h incubation, the cells were washed with cold PBS, lysed with NADP/NADPH Extraction Buffer and then incubated at 60 °C in order to decompose the NADP particles. The final readings were made using the Power Wave Microplate Spectrophotometer (Bio‐Tek) at 450 nm. Each experiment was conducted three times with measurement in triplicate.

### Reduced glutathione level

Cells were inoculated into a six‐well plate at a concentration of 2.5 × 10^5^ cells·mL^−1^. The tested compounds were added when 70–80% confluence was achieved. After a 48‐h incubation, the cells were washed with PBS and detached using 0.25%/EDTA trypsin (Corning). Next, the cells were stained with a solution containing propidium iodide, acridine orange and VitaBright‐48™, which stains viable cells in an intensity‐dependent manner relying on their level of reduced thiols. Analysis was performed using image cytometry with the Nucleo Counter NC3000 (Chemometec). Each experiment was conducted three times with measurement in triplicate.

### Glutamine concentration in the cell culture medium

Glutamine concentration was determined using ion‐exchange chromatography in the Amino Acids Analyzer AAA 500 (INGOS Corp., Prague, Czech Republic). The cell culture media were deproteinized with 6% sulphosalicilic acid in a lithium‐citrate buffer (pH 2.6), and centrifuged at 15000 ***g*** for 20 min. Separation of free amino acids was performed on the analytic column OSTON LG FA (INGOS Corp.) and five lithium citrate buffers (pH 2.90, 3.10, 3.35, 4.05, 4.65). Evaluation was performed with clarity 6.1 software (Data Apex, Prague, Czech Republic). The quantity of glutamine was measured according to a linear standard curve. Glutamine consumption was normalized to cell number. Each experiment was conducted three times with measurement in triplicate.

### Statistical analysis

The results were statistically analysed with statistica v. 13 (StaftSoft, Krakow, Poland). The data were calculated as mean ± SD. In order to compare more than two groups, one‐way analysis of variance (ANOVA) and *post hoc* multiple comparisons with Tukey's HSD test were used. All parameters were considered statistically significantly different if *P* values were less than 0.05.

## Results

### Cytotoxicity analyses

**Figure 1:**
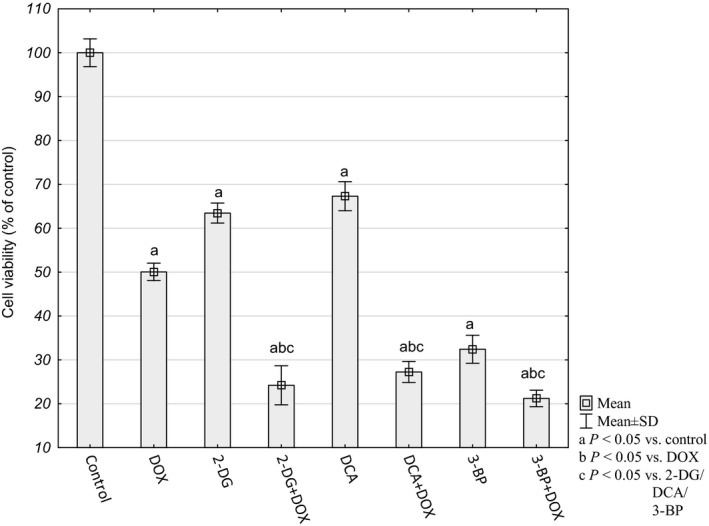
Relative HepG2 cell viability determined by MTT assay. The results were calculated as percentage of control cultures, which were averaged to define 100%. Values are presented as mean ± SD derived from three independent experiments. To compare more than two groups, one‐way ANOVA and *post hoc* multiple comparisons on the basis of Tukey's HSD test were used.

**Figure 2:**
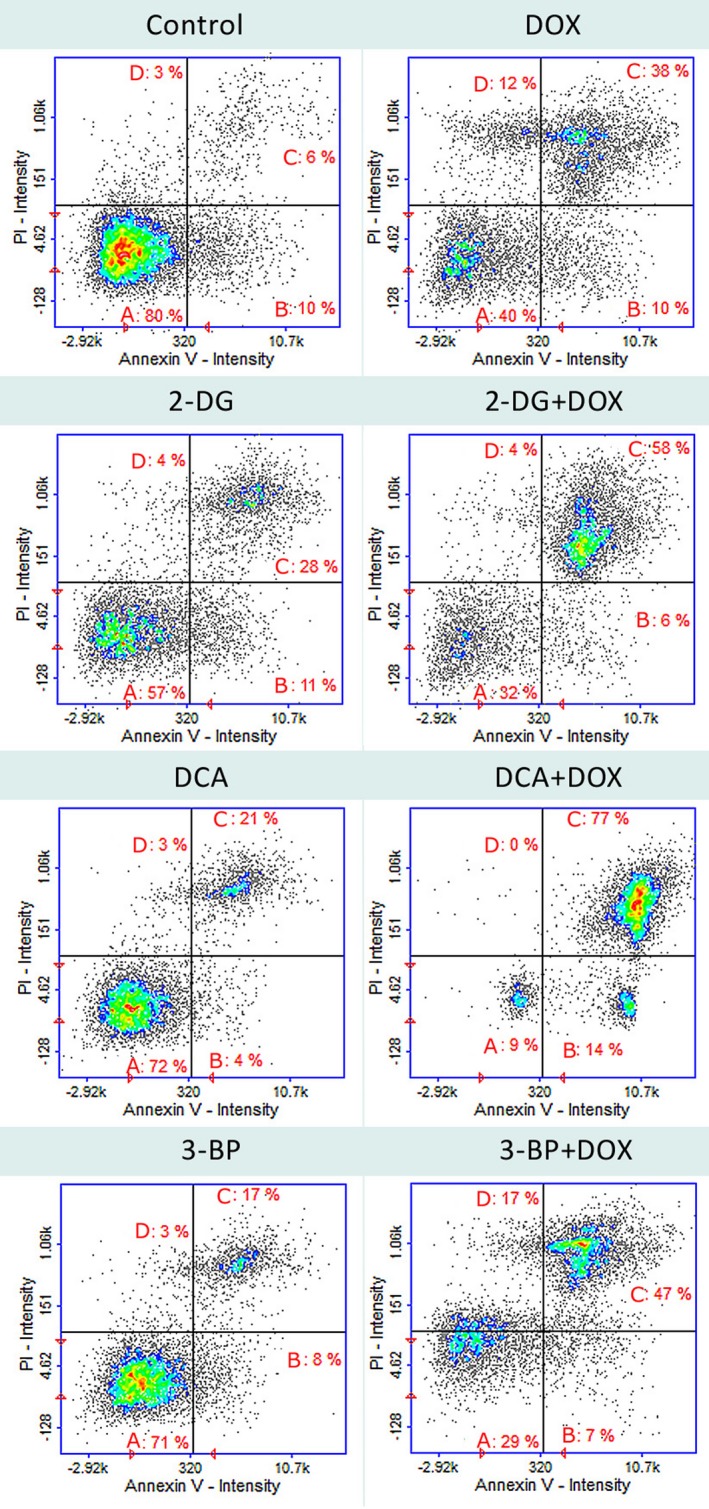
Cell apoptosis/necrosis of HepG2 cells, stained with annexin V–FITC and propidium iodide for image cytometry. (A) Live, (B) early apoptotic, (C) late apoptotic and (D) necrotic cells. The results show one representative experiment of three independently performed that showed similar patterns.

The MTT test revealed a moderate impact of 1 μm DOX on HepG2 cells (50.06 ± 1.98%). Concentrations of glycolysis inhibitors were chosen on the basis of our previous preliminary studies – 1 mm 2‐DG, 1 mm DCA and 1 μm 3‐BP were the lowest concentrations that significantly attenuated DOX toxicity against HepG2 cells. All the tested glycolysis inhibitors were cytotoxic for the HepG2 cells alone, and 3‐BP was more toxic than DOX (32.43 ± 3.19%). Simultaneous treatment with DOX had the strongest effect in the case of 2‐DG and 3‐BP (24.22 ± 4.44% and 21.22 ± 1.88%, respectively; Fig. 1). As Fig. 2 shows, the MTT test result was confirmed by the apoptosis/necrosis assay.

### mRNA expression analysis

First, using the qRT‐PCR, we evaluated the level of expression of selected genes related to energy metabolism: the glucose transporter (*GLUT1*), hexokinase 2 (*HK2*), lactate dehydrogenase A (*LDHA*), phosphoenolpyruvate carboxykinase 2 (*PCK2*), pyruvate dehydrogenase kinase 1 (*PDK1*), nicotinamide nucleotide transhydrogenase (*NNT*); and genes involved in antioxidant defense: superoxide dismutase (*SOD*) and glutathione peroxidase (*GPX*).

The study revealed that DOX significantly reduced the expression of all the tested genes (Table 2). Unexpectedly, the mRNA levels for antioxidant *SOD* and *GPX* were also very low. A clear synergistic effect for all the inhibitors and DOX was observed only in the *SOD* expression analysis. 2‐DG and 3‐BP also significantly affected the *GPX* expression in the cells treated with DOX.

Analysis of changes in the energy metabolism gene expression pattern revealed no similarities between individual inhibitors. In the cells treated simultaneously with DOX and one of the glycolysis inhibitors, no synergistic effect or strong impact of one compound was observed (Table 2).

**Table 2:**
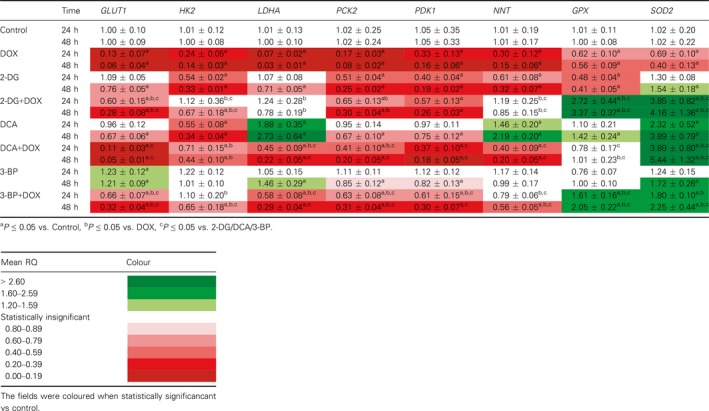
Relative mRNA expression level of selected genes related to energy metabolism and antioxidant defense. *RNA18SN5* and *PPIA* were used as reference genes. The results were calculated as RQ values and presented as mean ± SD. To compare more than two groups, one‐way ANOVA and *post hoc* multiple comparisons with Tukey's HSD test were used

### Oxidative stress

The mRNA expression analysis revealed a significant upregulation of the oxidative defense genes in cells treated simultaneously with DOX and one of the glycolyis inhibitors. For this reason, we evaluted oxidative stress markers.

**Figure 3:**
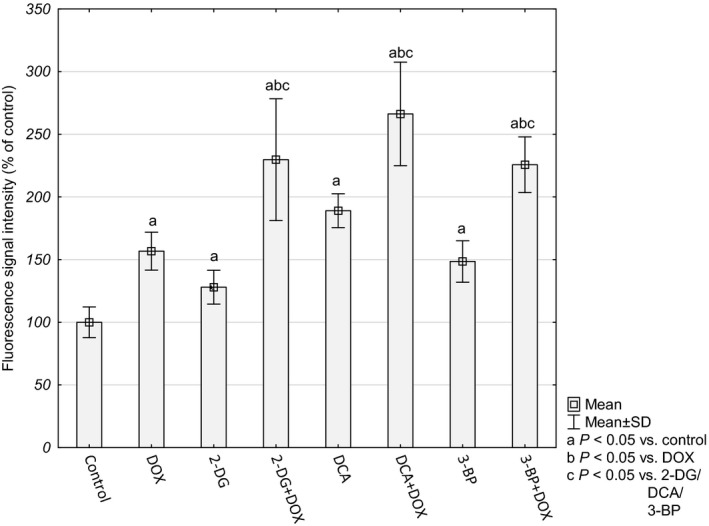
Mean fluorescence intensity of CellRox probe presented as percentage of fluorescence in control cultures, which were averaged to define 100%. Values are presented as mean ± SD derived from three independent experiments. To compare more than two groups, one‐way ANOVA and *post hoc* multiple comparisons with Tukey's HSD test were used.

Analysis of fluorescence intensity of the CellRox probe that depends on the presence of ROS revealed increased levels in all the tested cultures, but in the cells treated with both compounds it was significantly higher (Fig. 3).

**Figure 4:**
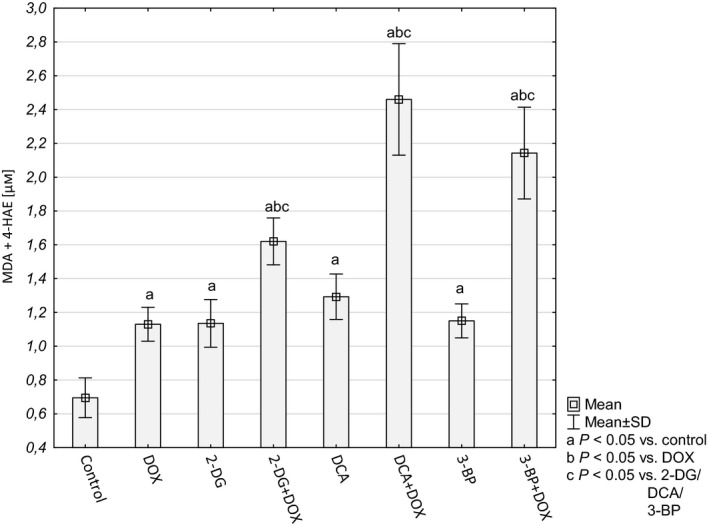
Lipid peroxidation level in HepG2 cells on the basis of MDA and 4‐HAE concentration (μm). Values are presented as mean ± SD derived from three independent experiments. To compare more than two groups, one‐way ANOVA and *post hoc* multiple comparisons with Tukey's HSD test were used.

In order to determine whether the increased amount of free radicals resulted in oxidative damage in the cells, we evaluated the concentration of the LPO products MDA and 4‐HAE. Increased LPO is crucial for cells dealing with an excess of ROS because this may result in the loss of intracellular and plasma membrane integrity. The MDA+4‐HAE concentration was elevated in all the tested cultures and, similarly to the level of ROS, the highest intensity of LPO was observed in the cells treated with both DOX and a particular glycolysis inhibitor (Fig. 4).

**Figure 5:**
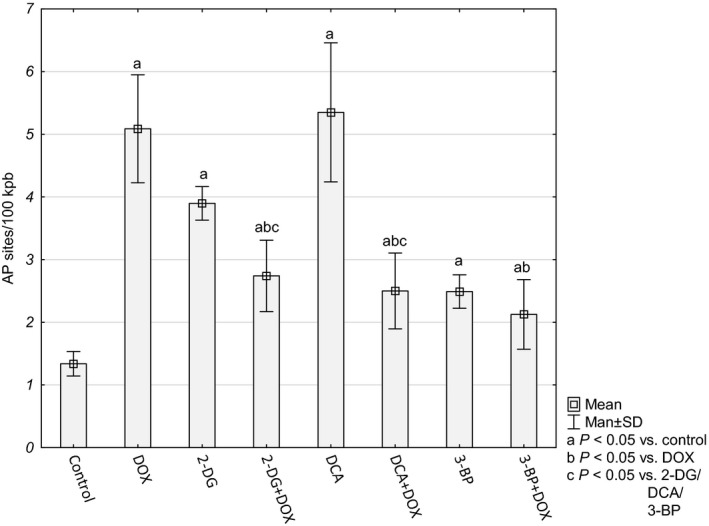
AP site number per 100 kpb in HepG2 cells. Values are presented as mean ± SD derived from three independent experiments. To compare more than two groups, one‐way ANOVA and *post hoc* multiple comparisons with Tukey's HSD test were used.

Formation of AP sites is one of the major types of damage generated by ROS (together with sugar modifications and strand breaks). In the tested cultures, the number of AP sites was higher in comparison to the control cells. The largest number of AP sites was observed for the DOX and DCA treatment (5.09 ± 0.86 and 5.35 ± 1.11 AP per 100 kbp, respectively). After combining DOX with 2‐DG or DCA, we observed a drop in the level of the AP sites in comparison to DOX alone or a particular inhibitor (Fig. 5). The number of AP sites in the DNA of the cells treated with 3‐BP+DOX and 3‐BP alone did not differ from each other (2.12 ± 56 and 2.49 ± 27 AP per 100 kbp) but was significantly lower than in the cells treated with DOX.

**Figure 6:**
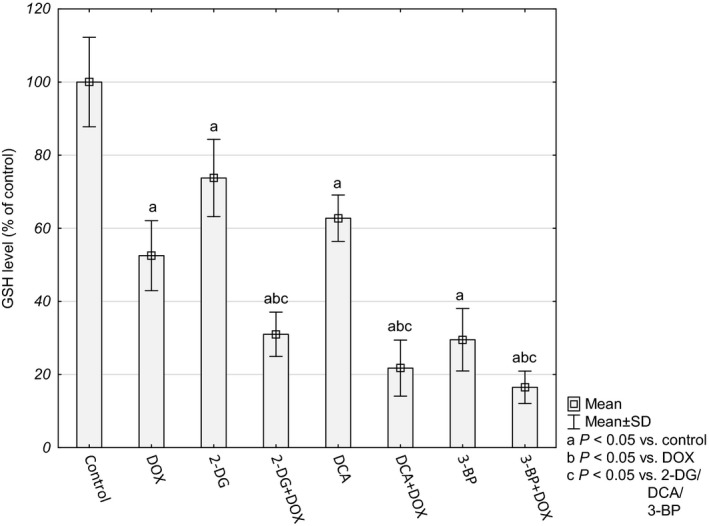
GSH level in HepG2 cells presented as percentage of control cultures, which were averaged to define 100%. Values are presented as mean ± SD derived from three independent experiments. To compare more than two groups, one‐way ANOVA and *post hoc* multiple comparisons with Tukey's HSD test were used.

**Figure 7:**
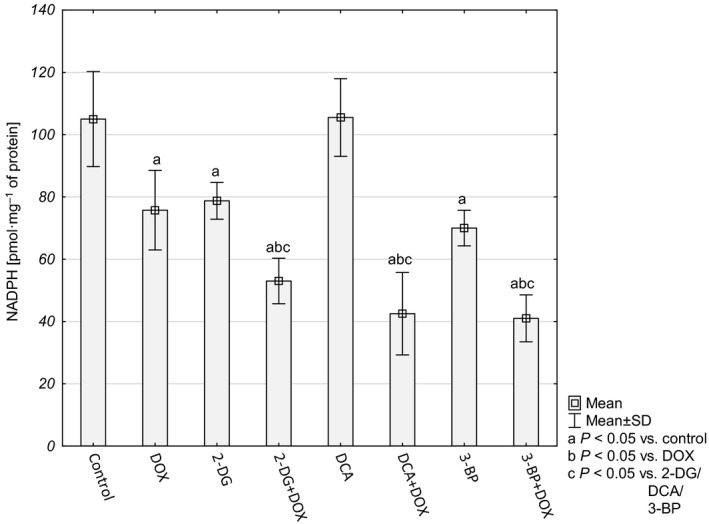
NADPH concentration in HepG2 cells (pmol·mg^−1^ of protein). Values were presented as mean ± SD derived from three independent experiments. To compare more than two groups, one‐way ANOVA and *post hoc* multiple comparisons with Tukey's HSD test were used.

The overall cellular antioxidative force was evaluated on the basis of the level of reduced glutathione (GSH) and NADPH. A significant decrease in GSH and in NADPH was observed for the cultures treated with combined compounds (Figs 6 and 7).

**Figure 8:**
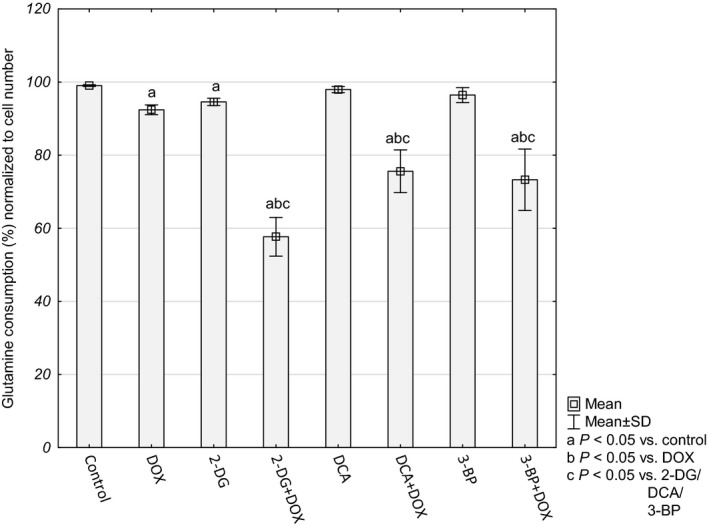
Glutamine consumption presented as percentage of control cultures and normalized to cell number. Values are presented as mean ± SD derived from three independent experiments. To compare more than two groups, one‐way ANOVA and *post hoc* multiple comparisons with Tukey's HSD test were used.

The presented results indicated that the cells probably had lost their ability to produce reductive force to glutathione regeneration and antioxidant defense. Inhibition of glycolysis on the level of phosphorylation of glucose to glucose‐6‐phosphate (HK inhibitors – 2‐DG and 3‐BP) deprives the cells of the substrate for the pentose phosphate pathway – the main source of NADPH and cellular building blocks. The other source of reductive force and biomass is the glutaminolysis pathway. Therefore, in order to check how the cells treated with the tested compounds made use of this source, we evaluated glutamine consumption. Interestingly, after 48 h the control cells as well as the cells incubated with a single compound (DOX or inhibitor) utilized almost 100% of glutamine contained in the culture medium, while the cells treated with a combination of DOX + an inhibitor used 57.67 ± 5.29%, 75.59 ± 5.84% and 73.27 ± 8.39% for 2‐DG+DOX, DCA+DOX and 3‐BP+DOX, respectively (Fig. 8). This seems to confirm the supposition that the tested cells decreased the possibility of reductive force production and antioxidant capacity.

## Discussion

Glycolysis inhibitors have been intensively studied in recent years as a group of compounds with a potential anticancer activity. This is associated with increasing knowledge of metabolic disorders in cancer cells. Otto Warburg discovered that the cells from most cancers are characterized by increased glucose uptake and exhibit a very intense sugar (glucose) consumption, up to 10‐fold higher than in normal cells 20. It should be stressed that in the light of many studies, ATP is not the goal of increased glycolysis, which is, after all, many times less efficient than OXPHOS 1, 3. In intensively proliferating cells it is important to redirect the uptake of glucose to the pathways of synthesis of biomass and reducing agents, such as the pentose phosphate pathway. It has also been hypothesized that a higher level of ROS production in cancer cells will result in an increased demand for reducing equivalents and intensified glucose metabolism 21. For this reason, glucose metabolism is integrally related to antioxidant defense. All this is consistent with the results of research into the mechanism of action of glycolysis inhibitors, where oxidative stress in the cells treated with this kind of compound is a common observation 22, 23, 24. Moreover, in the present study the treatment of cancer cells with all the tested inhibitors induced oxidative stress symptoms, such as LPO, oxidative DNA damage and decreased levels of GSH and NADPH. The mechanism of this phenomenon, however, is different for each compound.

2‐Deoxyglucose competes with glucose for uptake via glucose transporters and then for phosphorylation by HK at the entry point into glycolysis. It disrupts the NADP^+^/NADPH balance since the phosphorylated form of 2‐DG (2‐DG‐6‐P) can proceed only through the first step in the pentose cycle via glucose 6‐phosphate dehydrogenase, leading to the regeneration of one molecule of NADPH 21, 25. 3‐BP, which is an analog of lactic acid, is an inhibitor of HK II; however, it is also an alkylating agent and can affect a number of macromolecules in a non‐specific manner. It was revealed that 3‐BP influenced glycolytic enzymes downstream of HK as well as mitochondrial enzymes, leading to inhibition of ATP production in the intensively dividing cells 26, 27. DCA inhibits PDK, thereby activating the pyruvate dehydrogenase complex. Thus, DCA diverts metabolism from glycolysis back to oxidative phosphorylation contributing in this way to an increased mitochondrial ROS generation 28.

DOX is a compound that has revealed many mechanisms of action – the primary mechanism involves intercalation within DNA base pairs resulting in DNA strands breaks and inhibition of both DNA and RNA synthesis. 12. On the other hand, it generates the formation of ROS and oxidative stress. Several cell oxido‐reductases are able to univalently reduce the quinone moiety of DOX to a semiquinone radical. That is a highly unstable intermediate form so it can rapidly transfer an unpaired electron to molecular oxygen generating a superoxide anion. At the same time, the semiquinone form returns to the parent form of quinone. The superoxide anion radical initiates a cascade of free radical reactions whose products include hydrogen peroxide (H_2_O_2_) and a highly toxic hydroxyl radical (HO^·^) 29. Generation of ROS by DOX may also take place in a non‐enzymatic way, which results from the drug molecule's ability to chelate iron. In the formed complex, Fe^3+^ is reduced to Fe^2+^. The resulting compound is a free radical capable of transferring the electron to molecular oxygen to form a superoxide anion radical and thus initiate a cascade of free radical reactions 30.

**Figure 9:**
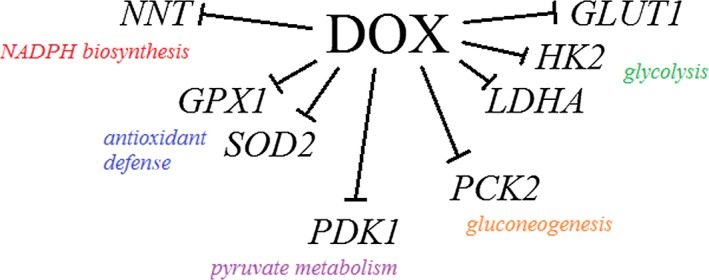
Summary of DOX inhibitory impact on tested genes’ expression. *GLUT1*, glucose transporter 1; *HK2*, hexokinase 2; *LDHA*, lactate dehydrogenase A; *PCK2*, phosphoenolpyruvate carboxykinase 2; *PDK1*, pyruvate dehydrogenase kinase 1; *NNT*, nicotinamide nucleotide transhydrogenase; *SOD2*, superoxide dismutase 2; *GPX1*, glutathione peroxidase 1.

The results of our study revealed that the interaction between DOX and glycolysis inhibitors is not just a simple summation of oxidative stress generated by both agents. We observed complex interactions at the level of oxido‐reductive balance that were closely related to disturbances in energy metabolism. First of all, in our study we noticed significantly downregulated expression of selected energy metabolism genes in the cells treated with DOX. This observation suggested that DOX alone may act as a glycolysis inhibitor – starting with glucose transport to the cell (*GLUT1*) and the first phosphorylation step (*HK2*), then *LDHA* – catalyzing the conversion of lactate to pyruvate and at the same time regenerating NAD^+^ from NADH, necessary to sustain glycolysis. We also observed a significant decrease in mRNA expression for *PCK2* – the enzyme that catalyzes the conversion of oxaloacetate to phosphoenolpyruvate, the rate‐limiting step in the metabolic pathway that produces glucose from lactate and other precursors derived from the citric acid cycle. As a result, a decreased PCK2 activity may impede the survival of tumor cells when the glucose level has been reduced 31. What is more, DOX was also found to reduce the *PDK1* expression, which, similarly to the DCA action, may switch metabolism from glycolysis to OXPHOS. The last evaluated gene that was significantly affected by DOX was *NNT* – the enzyme using the energy of the mitochondrial proton gradient to produce NADPH, transferring the reducing equivalent between NAD(H) and NADP(^+^). Unexpectedly, the expression of genes related to antioxidant defense was found to be significantly reduced (see the summary in Fig. 9).

It is quite likely that this global mRNA expression downregulation is the result of DOX intercalation to DNA. Drug interactions with DNA interfere with processes such as replication and transcription through an influence on DNA‐binding proteins, first of all on topoisomerase II but also on transcription factors 32. Interestingly, Yang *et al*. 33 observed that DOX induced DNA double‐strand breaks which occurred preferentially around promoters and correlated with the gene expression level.

Surprisingly, this effect was partially weakened and expression upregulated when the cells were simultaneously treated with one of the tested glycolysis inhibitors. We can only suppose that there might have occurred some disturbances either in DOX transport to the nucleus or during direct interactions with DNA. A reasonable explanation would be a lowered level of ATP under these circumstances – Kiyomiya *et al*. 34 found that DOX forms a complex with the proteasome and in this form it is transported to the nucleus in an ATP‐dependent manner. In our study, we observed that the cells treated with a single compound (DOX or an inhibitor) presented an elevated number of AP sites in DNA. However, simultaneous treatment surprisingly decreased the level of DNA damage, which is consistent with the hypothesis that the transport of DOX to the nuclei was restricted.

Regardless of disturbances in mRNA expression and DNA damage, a significantly lowered level of NADPH and GSH, the main antioxidative force, was the common phenomenon that accompanied the synergistic effect of DOX and a glycolysis inhibitor. Besides glucose, glutamine is another important source of energy, substrate synthesis and antioxidant defense. It is an important source of carbon and nitrogen for the cell and is significantly involved in GSH synthesis. Indirectly, it can also be a source of NADPH. Next to an increased utilization of glucose, which ‘produces’ lactate through the glycolytic pathway, an enhanced consumption of glutamine is the second hallmark of cancer cells 1.

**Figure 10:**
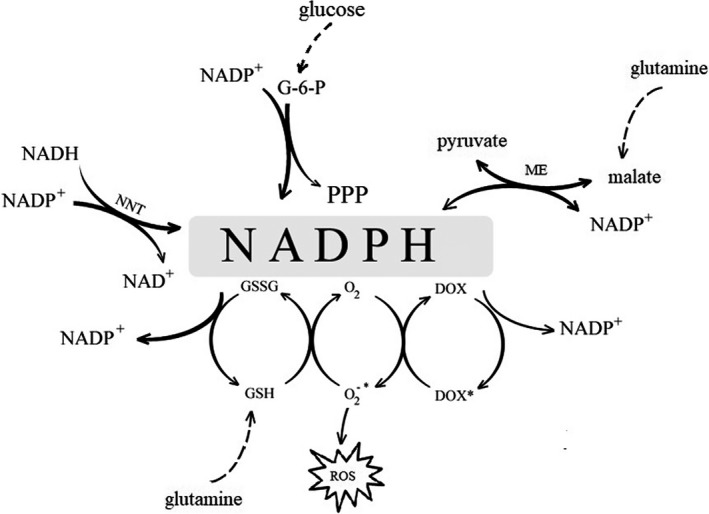
NADPH cell sources influenced by DOX in conditions of glycolysis inhibition.

For this reason, we investigated how intensively the cells consumed this amino acid in the conditions of impaired glucose metabolism and associated weakened antioxidant defense (low GSH and NADPH levels). There are a few contradictory reports about the possibility of utilization of glutamine by cancer cells while glucose metabolism is suppressed 35, 36, 37. In the present study inhibition of glycolysis by 2‐DG, 3‐BP and DCA did not change glutamine consumption in comparison to the control cultures – within 48 h almost all the content of this amino acid in the culture media was consumed. However, when the cells were simultaneously incubated with DOX, this uptake was significantly restricted. The mechanisms of dependency of glycolysis and glutaminolysis seem to be multifactorial and remain controversial 38. Our results seem to be in agreement with the study of Murai *et al*. 39 in which the authors stated that cancer cells in glucose‐restricted conditions become dependent on glutamine and malic enzyme for the supply of NADPH and pyruvate. In our study, the HepG2 cells had a reduced ability to take up glutamine only when both DOX and a glycolysis inhibitor acted together and, interestingly, regardless of the type of inhibitor and its particular mechanism of action. As a result of this, the cells lost the important alternative source of antioxidant defense. The most important sources of NADPH influenced by DOX under the conditions of glycolysis inhibition are summarized in Fig. 10.

## Conclusions

The obtained results revealed that DOX caused serious disturbances in the mRNA expression of energy metabolism and antioxidant defense genes. Both chemotherapeutic agents and glycolysis inhibitors induced oxidative stress in the HepG2 cells and associated damage. However, simultaneous treatment with two factors led to an even more intense LPO and a significant reduction in the GSH and NADPH levels. Disturbances in energy metabolism, including a reduced glutamine utilization, led to restrictions in NADPH production and GSH regeneration. Considering the potential use of glycolysis inhibitors to sensitize tumor cells to DOX as well as the presented results, further research must investigate the effect of glycolysis inhibitors on the development of anthracycline cardiotoxicity where oxidative stress plays the crucial role.

## Conflict of interest

The authors declare no conflict of interest.

## Author contributions

AK conceived and supervised the study and wrote the first draft of the manuscript, MO contributed to the formation of the experimental concept, carried out the research and revised the manuscript, MI conceived the study, designed the protocol and carried out the research, MH assisted in the research work and contributed to analysis of the data, and JD revised the manuscript.

## References

[1] DeBerardinis RJ , Lum JJ , Hatzivassiliou G and Thompson CB (2008) The biology of cancer: metabolic reprogramming fuels cell growth and proliferation. Cell Metab 7, 11–20. 18177721 10.1016/j.cmet.2007.10.002

[2] Koppenol WH , Bounds PL and Dang CV (2011) Otto Warburg's contributions to current concepts of cancer metabolism. Nat Rev Cancer 11, 325–337. 21508971 10.1038/nrc3038

[3] Upadhyay M , Samal J , Kandpal M , Singh OV and Vivekanandan P (2013) The Warburg effect: insights from the past decade. Pharmacol Ther 137, 318–330. 23159371 10.1016/j.pharmthera.2012.11.003

[4] Gambhir SS (2002) Molecular imaging of cancer with positron emission tomography. Nat Rev Cancer 2, 683–693. 12209157 10.1038/nrc882

[5] Kondoh H (2008) Cellular life span and the Warburg effect. Exp Cell Res 314, 1923–1928. 18410925 10.1016/j.yexcr.2008.03.007

[6] Buchakjian MR and Kornbluth S (2010) The engine driving the ship: metabolic steering of cell proliferation and death. Nat Rev Mol Cell Biol 11, 715–727. 20861880 10.1038/nrm2972

[7] Cairns RA , Harris IS and Mak TW (2011) Regulation of cancer cell metabolism. Nat Rev Cancer 11, 85–95. 21258394 10.1038/nrc2981

[8] Sheng H and Tang W (2016) Glycolysis inhibitors for anticancer therapy: a review of recent patents. Recent Pat Anticancer Drug Discov 11, 297–308. 27087655 10.2174/1574892811666160415160104

[9] Xu RH , Pelicano H , Zhou Y , Carew JS , Feng L , Bhalla KN , Keating MJ and Huang P (2005) Inhibition of glycolysis in cancer cells: a novel strategy to overcome drug resistance associated with mitochondrial respiratory defect and hypoxia. Cancer Res 65, 613–621. 15695406

[10] Nakano A , Tsuji D , Miki H , Cui Q , El Sayed SM , Ikegame A , Oda A , Amou H , Nakamura S , Harada T *et al* (2011) Glycolysis inhibition inactivates ABC transporters to restore drug sensitivity in malignant cells. PLoS One 6, e27222. 22073292 10.1371/journal.pone.0027222 PMC3206937

[11] Bean JF , Qiu YY , Yu S , Clark S , Chu F and Madonna MB (2014) Glycolysis inhibition and its effect in doxorubicin resistance in neuroblastoma. J Pediatr Surg 49, 981–984. 24888847 10.1016/j.jpedsurg.2014.01.037

[12] Agudelo D , Bourassa P , Bérubé G and Tajmir‐Riahi HA (2016) Review on the binding of anticancer drug doxorubicin with DNA and tRNA: structural models and antitumor activity. J Photochem Photobiol B 158, 274–279. 26971631 10.1016/j.jphotobiol.2016.02.032

[13] Singal PK , Li T , Kumar D , Danelisen I and Iliskovic N (2000) Adriamycin‐induced heart failure: mechanism and modulation. Mol Cell Biochem 207, 77–86. 10888230 10.1023/a:1007094214460

[14] Segredo MP , Salvadori DM , Roch NS , Moretto FC , Correa CR , Camargo EA , de Almeida DC , Reis RA , Freire CM , Braz MG *et al* (2014) Oxidative stress on cardiotoxicity after treatment with single and multiple doses of doxorubicin. Hum Exp Toxicol 33, 748–760. 24275640 10.1177/0960327113512342

[15] Oberley LW , Oberley TD and Buettner GR (1981) Cell division in normal and transformed cells: the possible role of superoxide and hydrogen peroxide. Med Hypotheses 7, 21–42. 6259499 10.1016/0306-9877(81)90018-9

[16] Sullivan LB and Chandel NS (2014) Mitochondrial reactive oxygen species and cancer. Cancer Metab 2, 17. 25671107 10.1186/2049-3002-2-17 PMC4323058

[17] Martinez‐Outschoorn UE , Trimmer C , Lin Z , Whitaker‐Menezes D , Chiavarina B , Zhou J , Wang C , Pavlides S , Martinez‐Cantarin MP , Capozza F *et al* (2010) Autophagy in cancer associated fibroblasts promotes tumor cell survival: role of hypoxia, HIF1 induction and NFκB activation in the tumor stromal microenvironment. Cell Cycle 9, 3515–3533. 20855962 10.4161/cc.9.17.12928 PMC3047617

[18] Sznarkowska A , Kostecka A , Meller K and Bielawski KP (2017) Inhibition of cancer antioxidant defense by natural compounds. Oncotarget 8, 15996–16016. 27911871 10.18632/oncotarget.13723 PMC5362541

[19] Gorrini C , Harris IS and Mak TW (2013) Modulation of oxidative stress as an anticancer strategy. Nat Rev Drug Discov 12, 931–947. 24287781 10.1038/nrd4002

[20] Warburg O , Wind F and Negelein E (1927) The metabolism of tumors in the body. J Gen Physiol 8, 519–530. 19872213 10.1085/jgp.8.6.519 PMC2140820

[21] Coleman MC , Asbury CR , Daniels D , Du J , Aykin‐Burns N , Smith BJ , Li L , Spitz DR and Cullen JJ (2007) 2‐deoxy‐D‐glucose causes cytotoxicity, oxidative stress, and radiosensitization in pancreatic cancer. Free Radic Biol Med 44, 322–331. 18215740 10.1016/j.freeradbiomed.2007.08.032

[22] Pułaski Ł , Jatczak‐Pawlik I , Sobalska‐Kwapis M , Strapagiel D , Bartosz G and Sadowska‐Bartosz I (2018) 3‐Bromopyruvate induces expression of antioxidant genes. Free Radic Res 26, 1–251. 10.1080/10715762.2018.1541176 30362385

[23] Saed GM , Fletcher NM , Jiang ZL , Abu‐Soud HM and Diamond MP (2011) Dichloroacetate induces apoptosis of epithelial ovarian cancer cells through a mechanism involving modulation of oxidative stress. Reprod Sci 18, 1253–1261. 21701041 10.1177/1933719111411731

[24] Shutt DC , O'Dorisio MS , Aykin‐Burns N and Spitz DR (2010) 2‐Deoxy‐D‐glucose induces oxidative stress and cell killing in human neuroblastoma cells. Cancer Biol Ther 9, 853–861. 20364116 10.4161/cbt.9.11.11632 PMC3215774

[25] Suzuki M , O'Dea JD , Suzuki T and Agar NS (1983) 2‐Deoxyglucose as a substrate for glutathione regeneration in human and ruminant red blood cells. Comp Biochem Physiol B 75, 195–197. 6872511 10.1016/0305-0491(83)90312-7

[26] Kim JS , Ahn KJ , Kim JA , Kim HM , Lee JD , Lee JM , Kim SJ and Park JH (2008) Role of reactive oxygen species‐mediated mitochondrial dysregulation in 3‐bromopyruvate induced cell death in hepatoma cells: ROS‐mediated cell death by 3‐BrPA. J Bioenerg Biomembr 40, 607–618. 19067133 10.1007/s10863-008-9188-0

[27] Shoshan MC (2012) 3‐Bromopyruvate: targets and outcomes. J Bioenerg Biomembr 44, 7–15. 22298255 10.1007/s10863-012-9419-2

[28] Ward NP , Poff AM , Koutnik AP and D'Agostino DP (2017) Complex I inhibition augments dichloroacetate cytotoxicity through enhancing oxidative stress in VM‐M3 glioblastoma cells. PLoS One 12, e0180061. 28644886 10.1371/journal.pone.0180061 PMC5482478

[29] Berthiaume JM and Wallace KB (2007) Adriamycin‐induced oxidative mitochondrial cardiotoxicity. Cell Biol Toxicol 23, 15–25. 17009097 10.1007/s10565-006-0140-y

[30] Zweier JL , Gianni L , Muindi J and Myers CE (1986) Differences in O_2_ reduction by the iron complexes of adriamycin and daunomycin: the importance of the sidechain hydroxyl group. Biochim Biophys Acta 884, 326–336. 2823890 10.1016/0304-4165(86)90181-9

[31] Leithner K , Hrzenjak A , Trötzmüller M , Moustafa T , Köfeler HC , Wohlkoenig C , Stacher E , Lindenmann J , Harris AL , Olschewski A *et al* (2015) PCK2 activation mediates an adaptive response to glucose depletion in lung cancer. Oncogene 34, 1044–1050. 24632615 10.1038/onc.2014.47

[32] Cutts SM , Parsons PG , Sturm RA and Phillips DR (1996) Adriamycin‐induced DNA adducts inhibit the DNA interactions of transcription factors and RNA polymerase. J Biol Chem 271, 5422–5429. 8621397 10.1074/jbc.271.10.5422

[33] Yang F , Kemp CJ and Henikoff S (2015) Anthracyclines induce double‐strand DNA breaks at active gene promoters. Mutat Res 773, 9–15. 25705119 10.1016/j.mrfmmm.2015.01.007 PMC4332850

[34] Kiyomiya K , Matsuo S and Kurebe M (2001) Mechanism of specific nuclear transport of adriamycin: the mode of nuclear translocation of adriamycin‐proteasome complex. Cancer Res 61, 2467–2471. 11289116

[35] Le A , Lane AN , Hamaker M , Bose S , Gouw A , Barbi J , Tsukamoto T , Rojas CJ , Slusher BS , Zhang H *et al* (2012) Glucose‐independent glutamine metabolism via TCA cycling for proliferation and survival in B cells. Cell Metab 15, 110–121. 22225880 10.1016/j.cmet.2011.12.009 PMC3345194

[36] Fan J , Kamphorst JJ , Mathew R , Chung MK , White E , Shlomi T and Rabinowitz JD (2013) Glutamine‐driven oxidative phosphorylation is a major ATP source in transformed mammalian cells in both normoxia and hypoxia. Mol Syst Biol 9, 712. 24301801 10.1038/msb.2013.65 PMC3882799

[37] Ganapathy‐Kanniappan S and Geschwind JF (2013) Tumor glycolysis as a target for cancer therapy: progress and prospects. Mol Cancer 12, 152. 24298908 10.1186/1476-4598-12-152 PMC4223729

[38] Wang L , Li JJ , Guo LY , Li P , Zhao Z , Zhou H and Di LJ (2018) Molecular link between glucose and glutamine consumption in cancer cells mediated by CtBP and SIRT4. Oncogenesis 7, 26. 29540733 10.1038/s41389-018-0036-8 PMC5852974

[39] Murai S , Ando A , Ebara S , Hirayama M , Satomi Y and Hara T (2017) Inhibition of malic enzyme 1 disrupts cellular metabolism and leads to vulnerability in cancer cells in glucose‐restricted conditions. Oncogenesis 6, e329. 28481367 10.1038/oncsis.2017.34 PMC5523067

